# The Mediating Role of Cardiorespiratory Fitness in the Association between a Negative Lifestyle and Poor Mental Health in Chilean Schoolchildren

**DOI:** 10.3390/children11070866

**Published:** 2024-07-17

**Authors:** Pedro Delgado-Floody, Felipe Caamaño-Navarrete, Guillermo Barahona-Fuentes, Carlos Arriagada-Hernández, Pablo Valdés-Badilla, Indya Del-Cuerpo, Mauricio Cresp-Barría, Manuel Gómez-López

**Affiliations:** 1 Department of Physical Education, Sport and Recreation, Universidad de La Frontera, Temuco 4811230, Chile; pedro.delgado@ufrontera.cl; 2 Physical Education Career, Universidad Autónoma de Chile, Temuco 4780000, Chile; felipe.caamano@uautonoma.cl (F.C.-N.); carlos.arriagada@uautonoma.cl (C.A.-H.); 3 Núcleo de Investigación en Salud Actividad Física y Deporte ISAFYD, Universidad de Las Américas, Sede Viña del Mar, Viña del Mar 2531098, Chile; g.barahonafuentes@uandresbello.edu; 4 Faculty of Education and Social Sciences, Universidad Andres Bello, Viña del Mar 2520000, Chile; 5 Grupo de Investigación Colaborativa para el Desarrollo Escolar (GICDE), Temuco 4780000, Chile; 6 Department of Physical Activity Sciences, Faculty of Education Sciences, Universidad Católica del Maule, Talca 3530000, Chile; pvaldes@ucm.cl; 7 Sports Coach Career, School of Education, Universidad Viña del Mar, Viña del Mar 2520000, Chile; 8 Department of Physical Education and Sport, Faculty of Sports Sciences, University of Granada, 18010 Granada, Spain; delcuerpo@ugr.es; 9 Strength & Conditioning Laboratory, CTS-642 Research Group, Department Physical Education and Sports, Faculty of Sport Sciences, University of Granada, 18010 Granada, Spain; 10 Department of Education and Innovation, Faculty of Education, Universidad Catolica de Temuco, Temuco 4780000, Chile; mcresp@uct.cl; 11 Department of Physical Activity and Sport, Faculty of Sport Sciences, University of Murcia, 30720 Murcia, Spain

**Keywords:** physical fitness, cardiorespiratory fitness, physical self-concept, depression, mental health, children

## Abstract

Background: A negative lifestyle has a reported relationship with psychological problems and deteriorated well-being. However, there is little information regarding the mediating role of cardiorespiratory fitness (CRF) in this relationship. Objectives: The objectives of the present study are twofold: first, to investigate the association between negative lifestyle, physical self-concept (PSC), and depression, and second, to assess the potential mediating role of CRF in this complex relationship. Methods: This cross-sectional study included 612 schoolchildren aged between 9 and 14 years from the Araucanía region (southern Chile). CRF was measured using the Leger test, and lifestyle, depression, and PSC were measured using validated questionnaires. Results: A negative lifestyle reported an inverse association with PSC (*p* < 0.001) and a positive association with depression levels (*p* < 0.001). The mediation analysis showed that CRF was positively related to PSC (*p* < 0.001) and inversely related to depression (*p* = 0.001); besides, the indirect effect CRF acted as a partial mediator in the association between a negative lifestyle and PSC (indirect effect = −1.15; SE = 0.01; 95% CI, −1.87, −0.55) and depression levels (indirect effect = 0.22; SE = 0.08; 95% CI, 0.08, 0.38). Conclusion: In conclusion, CRF in schoolchildren played a potential mediating role in the association between a negative lifestyle and depression and PSC.

## 1. Introduction

Mental health is a multidimensional state of subjective well-being. Some studies have reported an increase in mental health disorders in youths [1], including internalizing psychological disorders such as depression and anxiety and externalizing disorders such as conduct problems. In this sense, depression is defined as a mood condition, such as excessive sadness and/or a significantly reduced experience of pleasure. Children and adolescents have a global prevalence of 21.3% for mild-to-severe depression and 3.7% for major depression [2].

Additionally, social, cultural, and negative lifestyles that include high screen time per day (ST) and low physical activity per week (PA) may influence psychological disorders of mental health [3,4]. Therefore, addressing poor mental health among schoolchildren could be considered a public health priority. Existing evidence suggests that the school stage is a crucial period for the development of subjective well-being [5], where previous studies conducted among Chilean youths have reported low levels of subjective well-being [4,6]. Specifically, well-being is a complex and multifaceted concept that encompasses physical, psychological, and social dimensions [7,8].

Physical self-concept (PSC) is essential for mental health and well-being [9,10], and the perception of PSC is significantly correlated with health perception and psychological well-being [11]. Furthermore, higher subjective well-being among youths has been associated with various benefits, such as increased longevity, career success, and better personal relationships [12]. In this regard, a recent study indicated that PSC is negatively associated with anxiety [13]. Another study suggested that improving PSC through exercise participation can contribute to better mental well-being, highlighting PSC as a positive element of a happy life and self-esteem [14]. Indeed, existing research shows a general consensus that developing a positive self-concept is helpful for subjective well-being [15].

Previous data regarding children and young people have indicated an increase in the prevalence of depressive symptoms [16]. In fact, evidence has been reported that 34% of adolescents (aged 10–19 years) globally are at risk of developing clinical depression [17]. Depression is considered one of the most significant health problems, and it is well established that depression has negative consequences for people’s daily lives [18]. It has also been shown that a negative self-view can be linked with depressive symptoms [19], and patients with depression are characterized by low PSC [20]. Based on previously reported findings, strategies promoting PSC may have a beneficial impact on the treatment of depressive symptoms in young people [21].

Furthermore, studies have reported a strong association between negative lifestyle and poor mental and psychosocial well-being [22]. This negative behavior leads to the development of clinical and psychological problems in children [23]. The evidence has indicated that excessive ST for schoolchildren is associated with psychosocial problems, where more hours of ST are related to poorer psychological well-being [24], such as low self-esteem [25] and a high risk of increased mental health problems, including depression, anxiety, and dissatisfaction with school life [26] and poor PSC [6].

In addition, numerous studies have consistently provided compelling evidence demonstrating a robust association between a negative lifestyle and diminished mental health status [27]. Specifically, research indicates that inactive young individuals exhibit a higher prevalence of mental health issues, encompassing conditions such as mental disorders, depression, and anxiety [28]. Additionally, a separate investigation revealed that adolescents characterized by physical inactivity were significantly more prone to experiencing psychological distress, depressive disorders, and even bipolar II disorder when compared to their physically active counterparts [29]. Equally noteworthy, physical inactivity has been empirically linked to a reduced quality of life [30].

Intriguingly, a longitudinal study conducted among Norwegian students unveiled a negative correlation between the weekly hours spent on PA during ages 15–16 and emotional as well as peer-related problems at ages 18–19 [31]. In stark contrast, research conducted among European adolescents has reported that engaging in PA and participating in sports are associated with enhanced subjective well-being and reduced levels of anxiety and depressive symptoms [32]. Moreover, a study involving students indicated a direct link between low levels of PA and heightened stress and anxiety problems [33]. Similarly, another investigation found that a lower level of PSC was prevalent among individuals who engaged in PA [34]. Notably, PA may wield a positive influence on PSC among children and adolescents [35].

It is paramount to acknowledge the complexity of these issues, as poor mental and psychosocial well-being have been found to intertwine with compromised academic performance, impaired social interaction, and familial dysfunction [36,37,38]. Within this context, cardiorespiratory fitness (CRF), as measured by VO_2max_, emerges not only as a robust health marker but also as an attribute closely associated with optimism and overall well-being [39]. Substantiated by compelling evidence, a positive relationship between CRF and mental health outcomes, as well as general well-being, has been well-documented [40,41]. Moreover, individuals with higher levels have exhibited a reduced incidence of anxiety and depression symptoms [42]. A recent study underscored the correlation between a subpar PSC and low CRF in schoolchildren [6]. Furthermore, a positive association between elevated CRF and superior mental and psychosocial well-being among youth has been reported [43]. Additionally, improved PSC may positively influence enjoyment and satisfaction in physical education classes [44], and physical education classes can effectively contribute to enhancing youths’ CRF [45].

Considering the aforementioned, the hypothesis posits that a stronger PSC among adolescents could promote healthy behaviors, such as physical activity. While there is evidence that CRF positively impacts mental health, specific aspects remain unexplored. Particularly, it is unclear how CRF precisely mediates the relationship between an adverse lifestyle and overall psychological well-being in adolescents. This lack of detailed understanding limits our ability to design effective interventions that could enhance both physical self-concept and mental health in this vulnerable population. Therefore, it is crucial to further investigate these underlying mechanisms to develop more precise and effective health promotion strategies among adolescents. Therefore, the objectives of the present study are twofold: first, to investigate the association between an adverse lifestyle and both PSC and levels of depression, and second, to assess the potential mediating role of CRF in this complex relationship.

## 2. Materials and Methods

### 2.1. Participants

In this cross-sectional study, we enrolled schoolchildren aged between 9 and 14 years old (*n* = 612; *n*_girls_ = 262) from public and subsidized schools in southern Chile during 2023. The sample was intentional and non-probabilistic.

Participation in the study was contingent upon obtaining signed assent from the schoolchildren themselves, as well as informed consent from their respective parents or guardians. The research adhered to the principles outlined in the Helsinki Declaration (2013) and received approval from the Ethics Committee of Universidad Autónoma de Chile, Chile (ACTA; N° CEC 11–23).

The inclusion criteria encompassed the following conditions: (i) obtaining informed consent from both parents and assent from the participating schoolchild, (ii) enrolment in educational institutions, and (iii) being within the age range of 9 to 14 years. Exclusion criteria were as follows: (i) any musculoskeletal injuries or medical contraindications (i.e., congenital heart disease, fever, diarrhea, or general malaise) that would prevent their average performance in the assessments. Furthermore, children with permanent educational needs mentioned in Decree No.83 of the Chilean Ministry of Education [46], such as visual, hearing, intellectual, or multiple disabilities, dysphasia, or autistic disorder, were excluded from participation in this study.

### 2.2. Main Outcomes

#### 2.2.1. Lifestyle

Lifestyle assessment was conducted utilizing the PA Krece Plus test as outlined in previous research [47]. This assessment involved the classification of a child’s lifestyle based on daily hours dedicated to activities such as watching television or playing video games (ST) and the amount of PA engaged in after-school hours on a weekly basis. Classification was performed by computing the average for each of these two parameters.

To determine the overall lifestyle classification, the total points assigned for ST were first inverted and then combined with the points allocated for PA after school hours. Based on the resultant combined score, individuals were categorized into one of three lifestyle groups: (i) “Good lifestyle” (for boys with ≥9 h and girls with ≥8 h of combined ST and PA), (ii) “Regular lifestyle” (for boys with 6–8 h and girls with 5–7 h), or (iii) “Poor lifestyle” (for boys with ≤5 h and girls with ≤4 h). The questionnaires used for this assessment were administered individually to the children in the presence of the researchers to ensure the accuracy and consistency of responses.

#### 2.2.2. Cardiorespiratory Fitness

CRF assessment was conducted utilizing the progressive 20 m shuttle run test [48]. During this test, participants were instructed to run between two lines positioned 20 m apart while synchronizing their pace with audio signals emitted from a pre-recorded CD. It is noteworthy that this test has undergone validation specifically among Chilean schoolchildren and has been previously employed in the context of the Physical Education National Study. To ensure consistency and comparability of results, the outcomes of the 20 m shuttle run test were standardized according to the protocol established by Leger. Subsequently, the calculation of VO_2max_ was performed employing Leger’s equation [48].

#### 2.2.3. Depression

The Children’s Depression Inventory (CDI) was employed to assess depression levels in the participants [49]. It was adapted into Spanish by Barrio et al. [50]. This questionnaire comprises 27 sets of three statements that pertain to various aspects of depressive symptomatology. Respondents are provided with three response options: 0, indicating the absence of symptoms; 1, indicating the presence of mild symptoms; and 2, indicating the presence of definite symptoms. The cumulative score generated from the questionnaire can range from 0 to 54, with scores exceeding 18 points indicating a potential presence of depression. It is important to note that higher scores on the CDI are indicative of more pronounced levels of depression.

#### 2.2.4. Self-Concept

The PSC Questionnaire (CAF) was utilized for our study. This questionnaire has psychometric properties that make it suitable for application from preadolescence onwards [51,52]. It consists of a total of 36 items, of which 20 are presented directly and 16 in an inverse manner. Participants evaluate these items using a 5-point Likert-type scale, where a score of 1 represents “false” and 5 signifies “true”. The PSC is divided into six distinct dimensions:

(i) “Physical ability”, in which students express their thoughts concerning their sporting abilities, such as “I look clumsy in sports activities.” This dimension represents one’s self-perception regarding their ability to engage in sports;
(ii) “Physical condition” assesses ideas like “I have a lot of physical energy” or “I can engage in prolonged physical activity without becoming tired.” It pertains to confidence in one’s physical condition and the perception of one’s resistance to engaging in strenuous physical activities;
(iii) “Physical appearance” incorporates expressions like “I find it difficult to maintain a good physical appearance” or “I feel confident about the physical image I project”. This dimension pertains to one’s perception of their physical appearance and their level of satisfaction with the image they present to others;
(iv) “Strength” assesses ideas such as “I am capable of performing activities that require strength” or “I am strong”. It relates to one’s self-perceived strength and their ability to perform activities that demand physical power, such as lifting weights;
(v) “General PSC” involves statements like “Physically, I am satisfied with myself” or “I feel less capable than others”. This dimension represents overall opinions and emotions, including happiness, satisfaction, pride, and confidence, in the physical domain;
(vi) “General subscale self-concept” includes expressions such as “I feel happy” or “I wish I were different”. This dimension evaluates subjects’ satisfaction levels with themselves and their life in general terms.

The CAF questionnaire provides a comprehensive assessment of various facets of PSC, encompassing both specific attributes and general well-being.

#### 2.2.5. Anthropometric Parameters

To assess the body mass (kg) of the children in the study, a TANITA scale model was employed, Scale Plus UM-028 (Tokyo, Japan). The measurements were taken with the children wearing only their underclothes and without any footwear. For the estimation of their height (m), a Seca^®^ stadiometer model 214 (Hamburg, Germany), which is graduated in millimeters (mm), was utilized.

Subsequently, the Body Mass Index (BMI) for each participant was calculated. BMI was computed as the individual’s body mass (kg) divided by the square of their height in meters (kg/m^2^) [53]. This standard method provides a valuable indicator of the participant’s body composition and is widely used in clinical and research settings to assess health and nutritional status.

### 2.3. Procedure

Research assistants visited during physical education class day at school. The data were collected over three separate sessions by a team of trained researchers. Physical fitness was evaluated in the first session. In the second session, anthropometric assessments were carried out in a favorable space facilitated by the school with optimum temperature. Finally, lifestyle surveys and well-being instruments were applied in the classrooms and in the presence of researchers (helped with any potential questions).

### 2.4. Statistical Analysis

Normal distribution was tested using the Kolmogorov–Smirnov test. For continuous variables, values are presented at the mean and standard deviation (SD). Differences between mean values according to sex were determined using the ANOVA and the chi-square test, respectively. The association of PSC and depression with negative lifestyle and CRF was estimated with a simple linear regression adjusted by sex and age. To verify the effect of the mediating variables CRF (M), regression analyses were performed, considering negative lifestyle as an independent variable (X) and PSC and depression as dependent variables (Y). Within the analysis, the total effect (c), direct effect (c′), and indirect effect (a × b, IE) were calculated for the samples as well as the 95% confidence interval (CI) using the macro/interface process v. 3.3 for SPSS v. 23 and the bootstrapping method with a resampling rate of 5000 [54]. All the statistical analyses were performed with SPSS statistical software version 23.0 (SPSS^TM^ Inc., Chicago, IL, USA). The alpha level was set at *p* < 0.05 for statistical significance.

## 3. Results

Table 1 displays a comparison of study variables according to lifestyle categories (good vs. regular vs. bad). Significant differences were observed in CRF, general PSC, subscale self-concept, and depression. Specifically, the bad lifestyle group reported lower values in these variables compared to the good and regular lifestyle groups.

In terms of the PSC dimension, CRF exhibited positive associations with physical ability (β: 0.04, 95% CI: −0.01 to 0.09, *p* < 0.001), physical appearance (β: 0.07, 95% CI: 0.01 to 0.13, *p* = 0.025), strength dimension (β: 0.15, 95% CI: 0.09 to 0.21, *p* < 0.001), general PSC (β: 0.17, 95% CI: 0.11 to 0.24, *p* < 0.001), subscale PSC (β: 0.12, 95% CI: 0.07 to 0.18, *p* < 0.001), and the overall PSC score (β: 0.69, 95% CI: 0.49 to 0.88, *p* < 0.001). Additionally, CRF was negatively associated with depression (β: −0.15, 95% CI: −0.22 to −0.08, *p* < 0.001). These significant associations remained even after adjusting for sex and age. In relation to a negative lifestyle, there was an inverse association with strength dimension (β: −0.20, 95% CI: −0.38 to −0.01, *p* = 0.034), general PSC (β: −0.74, 95% CI: −0.93 to −0.55, *p* < 0.001), and the total PSC score (β: −1.40, 95% CI: −1.99 to −0.80, *p* < 0.001). Conversely, a negative lifestyle was positively associated with depression levels (β: 0.72, 95% CI: 0.51 to 0.92, *p* < 0.001). These significant associations remained robust after adjusting for sex and age (Table 2).

**Figure 1:**
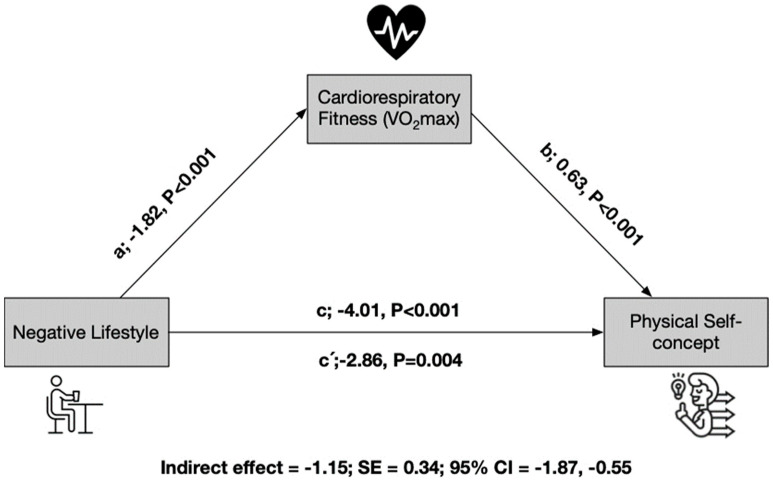
Mediations model testing whether the association between negative lifestyle and negative physical self-concept was mediated by cardiorespiratory fitness.

Figure 1 displays the results of the mediation analysis for the entire sample, consisting of 617 schoolchildren. In this analysis, CRF emerges as a mediating variable in the relationship between a negative lifestyle and PSC. In the first regression step (a), a negative lifestyle was found to have an inverse relationship with PSC (*p* < 0.001). In the second step, the regression coefficient of lifestyle in PSC remained significant (*p* = 0.004). In the third step, the potential mediator CRF was positively related to the dependent variable PSC *(p* < 0.001). Even when both lifestyle and CRF were included in the model, the regression coefficient remained statistically significant (*p* < 0.001). Finally, the indirect effect analysis confirms that CRF serves as a partial mediator of PSC, with an indirect effect of −1.15 and a standard error (SE) of 0.34. The 95% confidence interval for the indirect effect ranges from −1.87 to −0.55 (Figure 1).

**Figure 2:**
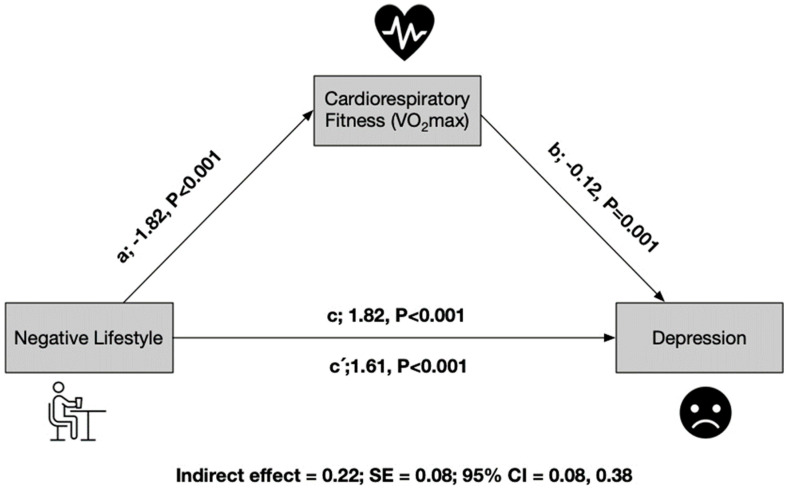
Mediations model testing whether the association between negative lifestyle and depression was mediated by cardiorespiratory fitness.

In Figure 2, CRF is shown to act as a mediating variable in the relationship between a negative lifestyle and depression. The mediation analysis proceeded through several steps: In the first regression step (a), a negative lifestyle was found to have an inverse relationship with CRF (*p* < 0.001), indicating that a negative lifestyle is associated with lower levels of CRF. In the second step, the regression coefficient of lifestyle in depression was also significant (*p* < 0.001), suggesting that a negative lifestyle is associated with higher levels of depression. In the third step, the potential mediator CRF was positively related to the dependent variable, depression (*p* = 0.001). This indicates that higher levels of CRF are associated with lower levels of depression. Importantly, even when both lifestyle and CRF were included in the model, the regression coefficient remained statistically significant (*p* < 0.001), indicating that both lifestyle and CRF independently contribute to depression. Finally, the indirect effect analysis confirms that CRF serves as a partial mediator of depression. The indirect effect has a value of 0.22 with a standard error (SE) of 0.08. The 95% confidence interval for the indirect effect ranges from 0.08 to 0.38 (Figure 2). This suggests that part of the relationship between a negative lifestyle and depression is mediated by CRF, highlighting the potential positive impact of improving CRF on reducing depression levels.

## 4. Discussion

The objectives of the present study are twofold: first, to investigate the association between an adverse lifestyle and both PSC and levels of depression, and second, to assess the potential mediating role of CRF in this complex relationship. The main results of this study are as follows: (i) a negative lifestyle is inversely associated with PSC and positively associated with depression levels; (ii) the mediation analysis showed that CRF was positively related to PSC and inversely related to depression (*p* = 0.001); and (iii) the indirect effect confirms that CRF is a partial mediator in the association between a negative lifestyle and PSC and depression levels.

Negative lifestyle was inversely linked to PSC (i.e., total score) and various dimensions of the PSC. Several studies have highlighted the detrimental role of a negative lifestyle in adolescents’ subjective well-being [4,55]. Moreover, a systematic review focusing on HRQoL and children’s lifestyle (i.e., PA and low ST) highlighted a negative relationship between ST and HRQoL, as well as lower psychological well-being [56]. In fact, existing evidence suggests that the negative impact of high ST on subjective well-being could be mitigated by high PA [57]. Another study indicated that improvements in physical self-perceptions can positively affect mental health [58]. Other evidence has suggested that prolonged ST has a negative impact on depression and anxiety scores [59] and subjective well-being [60].

A healthy lifestyle has been positively associated with PSC in students [34,35]. Indeed, PA has been proven to be a valuable tool for enhancing subjective well-being [61]. In this context, a recent study conducted among Chilean schoolchildren demonstrated that healthy lifestyle dimensions, such as PA after school and better nutritional habits, were positively correlated with PSC [6]. Similarly, a meditational model reported that PA had a direct effect on PSC and an indirect effect on general self-concept in Spanish adolescents; therefore, improving PA levels could be a promising strategy for promoting subjective well-being [9]. Moreover, previous evidence has indicated that adolescents with poorer PSC are at a higher risk of being physically inactive [62]. Additionally, data from Spanish students have shown that PA and diet quality are positively associated with PSC [34]. Based on previously reported findings, interventions promoting PA in schools can be beneficial for promoting the development of subjective well-being.

It has been suggested that a healthy lifestyle is associated with PSC and self-esteem among Chilean schoolchildren [63]. In this regard, a population-based study indicated that a negative lifestyle characterized by more than 4 h per day of ST was associated with poorer psychological well-being outcomes; furthermore, these relationships were stronger among adolescents than among children [24]. A cross-sectional study conducted with students reported that PSC dimensions were negatively correlated with ST and positively predicted levels of PA [64]. Similarly, a longitudinal study demonstrated the importance of physical self-perceptions in relation to PA patterns; high perceived competence increased the odds of being physically active by 3.8 times [65]. Evidence has also shown that a healthy lifestyle, including PA and the quality of one’s diet, improves individuals’ self-concept and body image perception [66]. Additionally, an ecological model described significant interpersonal barriers that influence a healthy lifestyle, such as PA patterns and dietary habits, including a lack of self-confidence and motivation [67].

It has also been shown that having higher levels of CRF may help improve levels of PSC and mental health in Spanish adolescents [68]. However, more longitudinal and interventional studies are needed to establish evidence of causation between physical fitness and mental health [69]. In the present study, schoolchildren’s CRF was found to be related to the dependent variables of depression and PSC, and it also appeared to have a potential mediating role in the relationship between a negative lifestyle and psychological well-being, specifically PSC and depression. This evidence contributes to solidifying the positive association between CRF and subjective well-being in youths [6,40]. Building on previously reported findings, it is suggested that during adolescence, depression may be linked to unhealthy behaviors, such as low levels of physical activity [70,71]. Similarly, it has been demonstrated that high CRF is associated with lower depressive symptoms [72]. In other words, good CRF can promote better mental health [73]. On the contrary, the evidence indicates that lower levels of CRF are associated with a greater risk of depression [74].

Recent studies have shown that fitness parameters, such as the muscle quality index, are related to better well-being in schoolchildren [75], and these fitness parameters play a mediating role in the relationship between a negative lifestyle, including sedentary time and abdominal obesity, and well-being, as measured by health-related quality of life [76]. Similarly, another observational study conducted with preschool children revealed that CRF acted as a mediating variable in the relationship between gross motor competence and well-being, especially physical well-being [77]. In this context, a recent study indicated that poor PSC was related to low CRF in Chilean students [6]. A previous systematic review and meta-analysis demonstrated a longitudinal association between CRF levels and mental health [78]. Another study among Spanish students reported that physical fitness, including CRF and handgrip strength, was positively associated with PSC [79]. Moreover, a cross-sectional study found that peak oxygen consumption was positively associated with all self-concept dimensions. Additionally, this investigation reported a negative association between body fat and PSQ in female adolescents [80]. Likewise, a study conducted with adolescent schoolchildren revealed that physical fitness, including the ALPHA-fitness test, and fatness mediated the association between lifestyle factors, such as physical activity, sedentary time, and Mediterranean diet adherence, and body dissatisfaction [81]. In this context, previous evidence indicated that cardiovascular endurance mediated the link between physical self-perception and physical activity in adolescents [82].

Although there is limited evidence of a causal relationship between improvements in CRF and mental health, a previous study reported that improvements in CRF could predict a greater reduction in the severity of depression [83]. Physical fitness seems to provide a buffer against stress-related diseases and the inflammatory process, as well as promoting increased positive mood and well-being [84]. Other evidence has suggested that the effects of inflammation on the brain may contribute to the development of various psychiatric disorders beyond depression [85].

### 4.1. Limitations

The main limitations of the present study were (i) the cross-sectional design, (ii) we used a convenience sample, (iii) CRF was not measured objectively, (iv) PA levels per week and ST per day were evaluated by a questionnaire and not measure objectively, (v) medical variables were not evaluated, and (vi) Cronbach’s alpha was not estimated.

Among the strengths, we could express (i) the use of culturally validated questionnaires ensures accurate data for Chilean adolescents, (ii) the simplicity of the assessments (which would allow their use and application in lifestyle interventions focused on youths), (iii) uniquely explore the mediating role of CRF between negative lifestyle and psychological well-being; therefore, they do not evaluate them independently as has commonly been done; and (iv) the findings support the inclusion of interventions aimed at improving CRF in school programs, promoting a holistic approach that addresses both the physical and mental health of adolescents in an integrated manner.

### 4.2. Practical Applications

The results suggest that school interventions should focus on improving CRF not only as a physical health goal but also as an integral strategy for enhancing psychological well-being and reducing depression. This research provides a foundation for developing school programs that integrate CRF improvement as a key component in addressing mental health issues, proposing a more holistic and multifaceted approach to health promotion among adolescents.

## 5. Conclusions

In conclusion, a negative lifestyle is inversely associated with PSC and positively associated with depression levels. Likewise, it has been demonstrated that CRF plays a potential mediating role in the relationship between a negative lifestyle and depression, as well as PSC in schoolchildren. Consequently, CRF emerges as a crucial factor for promoting the psychological well-being of children, and it should be considered in educational and healthcare settings to proactively address mental health issues. This study provides a novel perspective on an area that has been less investigated by exploring the mediating role of CRF between adverse lifestyle and psychological well-being. Consequently, CRF not only improves mental health but also has a potential mediating role between an adverse lifestyle and psychological well-being in schoolchildren.

## Figures and Tables

**Table 1:** Comparison of study variables according to lifestyle category.

	Good Lifestyle (*n* = 112) A	Regular Lifestyle (*n* = 239) B	Bad Lifestyle (*n* = 261) C	*p*-Value _(F-Value)_
Age (y)	11.75 ± 1.11	11.64 ± 1.04	11.66 ± 1.08	0.680 _(0.39)_
Body mass (kg)	52.14 ± 16.20	50.55 ± 12.68	54.20 ± 14.46	0.015 _(4.20)_
Size (m)	1.54 ± 0.11	1.54 ± 0.10	1.56 ± 0.11	0.107 _(2.24)_
BMI (kg/m^2^)	21.52 ± 5.03	21.08 ± 4.25	22.06 ± 4.71	0.056 _(2.89)_
VO_2max_ (mL/kg/min)	48.32 ± 9.36	45.74 ± 6.84	44.46 ± 6.56	*p* < 0.001 _(11.29)_
Physical self-concept (score)	134.74 ± 18.97	128.59 ± 17.05	126.09 ± 19.57	*p* < 0.001 _(8.69)_
Physical self-concept dimension				
✓ Physical ability (score)	22.21 ± 5.35	21.85 ± 4.83	22.18 ± 5.05	0.707 _(0.35)_
✓ Physical condition (score)	20.72 ± 5.94	19.81 ± 5.94	20.18 ± 5.66	0.383 _(0.96)_
✓ Physical appearance (score)	21.91 ± 5.52	21.80 ± 5.92	21.31 ± 5.95	0.540 _(0.62)_
✓ Strength (score)	20.24 ± 5.93	19.69 ± 5.73	19.11 ± 5.71	0.189 _(1.67)_
✓ General PSC (score)	25.48 ± 5.22	22.84 ± 5.78	21.02 ± 6.45	*p* < 0.001 _(22.67)_
✓ Subscale PSC (score)	24.18 ± 5.06	22.59 ± 5.55	22.29 ± 5.28	0.006 _(5.14)_
Depression (score)	11.46 ± 5.64	12.56 ± 5.94	14.89 ± 7.25	*p* < 0.001 _(14.06)_

The data are presented as means and standard deviations (M ± SD), with statistical significance set at *p* < 0.05. BMI = body max index. VO_2max_ = maximum oxygen consumption.

**Table 2:** Association between CRF and lifestyle with psychological variables.

	Cardiorespiratory Fitness (−) → (+)			*p*-Value	Negative Lifestyle (+) → (−)			*p*-Value
	β (95% CI)	Beta	SE		β (95% CI)	Beta	SE	
	**Physical Self-Concept Dimension**							
	Physical ability							
Model 0	0.04 (−0.01; 0.09)	0.06	0.03	0.137	−0.05 (−0.21; 0.11)	−0.02	0.08	0.551
Model 1	0.06 (0.00; 0.11)	0.08	0.03	0.056	−0.05 (−0.21; 0.11)	−0.03	0.08	0.536
	Physical condition							
Model 0	0.13 (0.07; 0.19)	0.17	0.03	*p* < 0.001		−0.04	0.10	0.382
Model 1	0.14 (0.07; 0.20)	0.17	0.03	*p* < 0.001	−0.08 (−0.27; 0.10)	−0.04	0.10	0.375
	Physical appearance dimension							
Model 0	0.07 (0.01; 0.13)	0.09	0.03	0.025	−0.15 (−0.34; 0.04)	−0.06	0.10	0.117
Model 1	0.09 (0.03; 0.16)	0.12	0.03	0.006	−0.15 (−0.34; 0.04)	−0.06	0.10	0.124
	Strength dimension							
Model 0	0.15 (0.09; 0.21)	0.19	0.03	*p* < 0.001	−0.20 (−0.38; −0.01)	−0.09	0.09	0.034
Model 1	0.15 (0.08; 0.21)	0.19	0.03	*p* < 0.001	−0.20 (−0.38; −0.01)	−0.08	0.09	0.036
	General physical self-concept							
Model 0	0.17 (0.11; 0.24)	0.21	0.03	*p* < 0.001	−0.74 (−0.93; −0.55)	−0.29	0.10	*p* < 0.001
Model 1	0.17 (0.10; 0.24)	0.21	0.04	*p* < 0.001	−0.74 (−0.93; 0.55)	−0.30	0.10	*p* < 0.001
	Subscale self-concept							
Model 0	0.12 (0.07; 0.18)	0.17	0.03	*p* < 0.001	−0.17 (−0.35; 0.00)	−0.08	0.09	0.051
Model 1	0.13 (0.07; 0.19)	0.18	0.03	*p* < 0.001	−0.17 (−0.35; 0.00)	−0.08	0.09	0.051
	Physical self-concept total							
Model 0	0.69 (0.49; 0.88)	0.27	0.10	*p* < 0.001	−1.40 (−1.99; −0.80)	−0.18	0.30	*p* < 0.001
Model 1	0.74 (0.54; 0.95)	0.29	0.11	*p* < 0.001	−1.40 (−1.99; −0.80)	−0.18	0.30	*p* < 0.001
	**Psychological Variable**							
	Depression							
Model 0	−0.15 (−0.22; −0.08)	−0.16	0.04	*p* < 0.001	0.72 (0.51; 0.92)	0.27	0.10	*p <* 0.001
Model 1	−0.15 (−0.22; −0.08)	−0.17	0.04	*p* < 0.001	0.72 (0.51; 0.92)	0.27	0.11	*p* < 0.001

The presented data represent beta coefficients with their corresponding 95% confidence intervals (95% CI). Two models were used: Model 0 (unadjusted) and Model 1 (adjusted for sex and age). (−) → (+) represents from smallest to largest; (+) → (−) represents from highest to lowest.

## Data Availability

The original contributions presented in the study are included in the article, further inquiries can be directed to the corresponding author.

## References

[1] Panchal U., Salazar de Pablo G., Franco M., Moreno C., Parellada M., Arango C., Fusar-Poli P. (2023). The impact of COVID-19 lockdown on child and adolescent mental health: Systematic review. Eur. Child. Adolesc. Psychiatry.

[2] Lu B., Lin L., Su X. (2024). Global burden of depression or depressive symptoms in children and adolescents: A systematic review and meta-analysis. J. Affect. Disord..

[3] Bor W., Dean A.J., Najman J., Hayatbakhsh R. (2014). Are child and adolescent mental health problems increasing in the 21st century? A systematic review. Aust. N. Z. J. Psychiatry.

[4] Caamaño-Navarrete F., Angel Latorre-Roman P., Guzmán-Guzmán I.P., Parraga Montilla J., Jerez-Mayorga D., Delgado-Floody P. (2022). Lifestyle mediates the relationship between self-esteem and health-related quality of life in Chilean schoolchildren. Psychol. Health Med..

[5] Vaquero-Solís M., Tapia-Serrano M.A., Hortigüela-Alcalá D., Sierra-Díaz M.J., Sánchez-Miguel P.A. (2021). Physical activity and quality of life in high school students: Proposals for improving the self-concept in physical education. Int. J. Environ. Res. Public. Health.

[6] Delgado-Floody P., Soto-García D., Caamaño-Navarrete F., Carter-Thuillier B., Guzmán-Guzmán I.P. (2022). Negative physical self-concept is associated to low cardiorespiratory fitness, negative lifestyle and poor mental health in Chilean Schoolchildren. Nutrients.

[7] Halbreich U. (2022). Well-being: Diversified perspectives in search of operational definitions. Int. J. Soc. Psychiatry.

[8] Schonhardt S., Sullivan S., Shisler Marshall R. (2023). A Focused Review of Multidimensional Well-Being Assessments. J. Wellness.

[9] Fernández-Bustos J.G., Infantes-Paniagua Á., Cuevas R., Contreras O.R. (2019). Effect of physical activity on self-concept: Theoretical model on the mediation of body image and physical self-concept in adolescents. Front. Psychol..

[10] Garn A.C., Morin A.J., White R.L., Owen K.B., Donley W., Lonsdale C. (2020). Moderate-to-vigorous physical activity as a predictor of changes in physical self-concept in adolescents. Health Psychol..

[11] Roh S.Y. (2018). The influence of physical self-perception of female college students participating in Pilates classes on perceived health state and psychological wellbeing. J. Exerc. Rehabil..

[12] Holder M.D. (2019). The contribution of food consumption to well-being. Ann. Nutr. Metab..

[13] Melguizo-Ibáñez E., Zurita-Ortega F., Ubago-Jiménez J.L., López-Gutiérrez C.J., González-Valero G. (2023). An explanatory model of the relationships between sport motivation, anxiety and physical and social self-concept in educational sciences students. Curr. Psychol..

[14] Kim I., Ahn J. (2021). The effect of changes in physical self-concept through participation in exercise on changes in self-esteem and mental well-being. Int. J. Environ. Res. Public. Health.

[15] Martín-Albo J., Núñez J.L., Domínguez E., León J., Tomás J.M. (2012). Relationships between intrinsic motivation, physical self-concept and satisfaction with life: A longitudinal study. J. Sports Sci..

[16] Keyes K.M., Hamilton A., Patrick M.E., Schulenberg J. (2020). Diverging trends in the relationship between binge drinking and depressive symptoms among adolescents in the US from 1991 through 2018. J. Adolesc. Health.

[17] Shorey S., Ng E.D., Wong C.H. (2022). Global prevalence of depression and elevated depressive symptoms among adolescents: A systematic review and meta-analysis. Br. J. Clin. Psychol..

[18] Bernardi L., Mattila M., Papageorgiou A., Rapeli L. (2023). Down but not yet out: Depression, political efficacy, and voting. Political Psychol..

[19] Hards E., Ellis J., Fisk J., Reynolds S. (2020). Negative view of the self and symptoms of depression in adolescents. J. Affect. Disord..

[20] Knapen J., Vermeersch J., Van Coppenolle H., Cuykx V., Pieters G., Peuskens J. (2007). The physical self-concept in patients with depressive and anxiety disorders. Int. J. Ther. Rehabil..

[21] Ames M.E., Leadbeater B.J. (2018). Depressive symptom trajectories and physical health: Persistence of problems from adolescence to young adulthood. J. Affect. Disord..

[22] Zhao J., Zhang Y., Jiang F., Ip P., Ho F.K.W., Zhang Y., Huang H. (2018). Excessive Screen Time and Psychosocial Well-Being: The Mediating Role of Body Mass Index, Sleep Duration, and Parent-Child Interaction. J. Pediatr..

[23] Domingues-Montanari S. (2017). Clinical and psychological effects of excessive screen time on children. J. Paediatr. Child. Health.

[24] Twenge J.M., Campbell W.K. (2018). Associations between screen time and lower psychological well-being among children and adolescents: Evidence from a population-based study. Prev. Med. Rep..

[25] Neophytou E., Manwell L.A., Eikelboom R. (2021). Effects of excessive screen time on neurodevelopment, learning, memory, mental health, and neurodegeneration: A scoping review. Int. J. Ment. Health Addict..

[26] Cao H., Qian Q., Weng T., Yuan C., Sun Y., Wang H., Tao F. (2011). Screen time, physical activity and mental health among urban adolescents in China. Prev. Med..

[27] Werneck A.O., Silva D.R., Malta D.C., Souza-Júnior P.R., Azevedo L.O., Barros M.B., Szwarcwald C.L. (2021). Physical inactivity and elevated TV-viewing reported changes during the COVID-19 pandemic are associated with mental health: A survey with 43,995 Brazilian adults. J. Psychosom. Res..

[28] Denche-Zamorano Á., Franco-García J.M., Carlos-Vivas J., Mendoza-Muñoz M., Pereira-Payo D., Pastor-Cisneros R., Merellano-Navarro E., Adsuar J.C. (2022). Increased risks of mental disorders: Youth with inactive physical activity. Healthcare.

[29] He J.-P., Paksarian D., Merikangas K.R. (2018). Physical activity and mental disorder among adolescents in the United States. J. Adolesc. Health.

[30] Pang J.C., Chan E.L., Lau H., Reeves K.K., Chung T.H., Hui H.W., Leung A.H., Fu A.C. (2023). The impacts of physical activity on psychological and behavioral problems, and changes in physical activity, sleep and quality of life during the COVID-19 pandemic in preschoolers, children, and adolescents: A systematic review and meta-analysis. Front. Pediatr..

[31] Sagatun A., Søgaard A.J., Bjertness E., Selmer R., Heyerdahl S. (2007). The association between weekly hours of physical activity and mental health: A three-year follow-up study of 15–16-year-old students in the city of Oslo, Norway. BMC Public. Health.

[32] McMahon E.M., Corcoran P., O’Regan G., Keeley H., Cannon M., Carli V., Wasserman C., Hadlaczky G., Sarchiapone M., Apter A. (2017). Physical activity in European adolescents and associations with anxiety, depression and well-being. Eur. Child. Adolesc. Psychiatry.

[33] Tajik E., Abd Latiff L., Adznam S.N., Awang H., Siew C.Y., Bakar A.A. (2017). A study on level of physical activity, depression, anxiety and stress symptoms among adolescents. J. Sports Med. Phys. Fit..

[34] Pérez-Mármol M., Chacón-Cuberos R., García-Mármol E., Castro-Sánchez M. (2021). Relationships among Physical Self-Concept, Physical Activity and Mediterranean Diet in Adolescents from the Province of Granada. Children.

[35] Babic M.J., Morgan P.J., Plotnikoff R.C., Lonsdale C., White R.L., Lubans D.R. (2014). Physical activity and physical self-concept in youth: Systematic review and meta-analysis. Sports Med..

[36] Barmola K. (2012). Family environment, mental health and academic performance of adolescents. Int. J. Sci. Res..

[37] Street J., Harris-Britt A., Walker-Barnes C. (2009). Examining relationships between ethnic identity, family environment, and psychological outcomes for African American adolescents. J. Child. Fam. Stud..

[38] Cooper K., Hards E., Moltrecht B., Reynolds S., Shum A., McElroy E., Loades M. (2021). Loneliness, social relationships, and mental health in adolescents during the COVID-19 pandemic. J. Affect. Disord..

[39] Rodriguez-Ayllon M., Cadenas-Sanchez C., Esteban-Cornejo I., Migueles J.H., Mora-Gonzalez J., Henriksson P., Martin-Matillas M., Mena-Molina A., Molina-Garcia P., Estevez-Lopez F. (2018). Physical fitness and psychological health in overweight/obese children: A cross-sectional study from the ActiveBrains project. J. Sci. Med. Sport..

[40] Janssen A., Leahy A.A., Diallo T.M., Smith J.J., Kennedy S.G., Eather N., Mavilidi M.F., Wagemakers A., Babic M.J., Lubans D.R. (2020). Cardiorespiratory fitness, muscular fitness and mental health in older adolescents: A multi-level cross-sectional analysis. Prev. Med..

[41] Raghuveer G., Hartz J., Lubans D.R., Takken T., Wiltz J.L., Mietus-Snyder M., Perak A.M., Baker-Smith C., Pietris N., Edwards N.M. (2020). Cardiorespiratory fitness in youth: An important marker of health: A scientific statement from the American Heart Association. Circulation.

[42] Hallgren M., Kandola A., Stubbs B., Wallin P., Andersson G., Ekblom-Bak E. (2020). Associations of exercise frequency and cardiorespiratory fitness with symptoms of depression and anxiety-a cross-sectional study of 36,595 adults. Ment. Health Phys. Act..

[43] Rodriguez-Solana A., Gracia-Marco L., Llorente-Cantarero F.J., Cadenas-Sanchez C., Marmol-Perez A., Gil-Cosano J.J., Moliner-Urdiales D., Ubago-Guisado E. (2023). Is higher physical fitness associated with better psychological health in young pediatric cancer survivors? A cross-sectional study from the i B one FIT project. Scand. J. Med. Sci. Sports.

[44] Morales-Sánchez V., Hernández-Martos J., Reigal R.E., Morillo-Baro J.P., Caballero-Cerbán M., Hernández-Mendo A. (2021). Physical Self-Concept and Motor Self-Efficacy Are Related to Satisfaction/Enjoyment and Boredom in Physical Education Classes. Sustainability.

[45] Peralta M., Henriques-Neto D., Gouveia É.R., Sardinha L.B., Marques A. (2020). Promoting health-related cardiorespiratory fitness in physical education: A systematic review. PLoS ONE.

[46] Ministerio de Educación de Chile (2015). Diversificación de la Enseñanza. Decreto N° 83/2015.

[47] Majem L.S., Barba L.R., Bartrina J.A., Rodrigo C.P., Santana P.S., Quintana L.P. (2003). Obesidad infantil y juvenil en España. Resultados del Estudio enKid (1998-2000). Med. Clin..

[48] Leger L.A., Mercier D., Gadoury C., Lambert J. (1988). The multistage 20 metre shuttle run test for aerobic fitness. J. Sports Sci..

[49] Kovacs M. (1985). The Children’s Depression, Inventory (CDI). Psychopharmacol. Bull..

[50] del Barrio M.V., Moreno-Rosset C., López-Martínez R. (1999). El Children’s Depression Inventory,(CDI: Kovacs, 1992). Su aplicación en población española. Clínica Y Salud.

[51] Goñi A., Ruiz de Azúa S., y Rodríguez A. (2006). Cuestionario de Autoconcepto Fzsico (CAF).

[52] Goñi Grandmontagne A., Ruiz de Azúa S., Liberal I. (2004). Propiedades psicométricas de un nuevo cuestionario para la medida del autoconcepto físico. Rev. De Psicol. Del Deporte.

[53] Karnik S., Kanekar A. (2012). Childhood obesity: A global public health crisis. Int. J. Prev. Med..

[54] Preacher k., Hayes A. (2004). SPSS and SAS procedures for estimating indirect effects in simple mediation models. Behav. Res. Methods Instrum. Comput..

[55] Delgado-Floody P., Caamaño-Navarrete F., Guzmán-Guzmán I.P., Jerez-Mayorga D., Martínez-Salazar C., Álvarez C. (2020). Food habits and screen time play a major role in the low health related to quality of life of ethnic ascendant schoolchildren. Nutrients.

[56] Suchert V., Hanewinkel R., Isensee B. (2015). Sedentary behavior and indicators of mental health in school-aged children and adolescents: A systematic review. Prev. Med..

[57] Motamed-Gorji N., Qorbani M., Nikkho F., Asadi M., Motlagh M.E., Safari O., Arefirad T., Asayesh H., Mohammadi R., Mansourian M. (2019). Association of screen time and physical activity with health-related quality of life in Iranian children and adolescents. Health Qual. Life Outcomes.

[58] Lubans D., Richards J., Hillman C., Faulkner G., Beauchamp M., Nilsson M., Kelly P., Smith J., Raine L., Biddle S. (2016). Physical activity for cognitive and mental health in youth: A systematic review of mechanisms. Pediatrics.

[59] Labrague L.J. (2014). Facebook use and adolescents’ emotional states of depression, anxiety, and stress. Health Sci. J..

[60] García-Hermoso A., Hormazábal-Aguayo I., Fernández-Vergara O., Olivares P.R., Oriol-Granado X. (2020). Physical activity, screen time and subjective well-being among children. Int. J. Clin. Health Psychol..

[61] Rodriguez-Ayllon M., Cadenas-Sánchez C., Estévez-López F., Muñoz N.E., Mora-Gonzalez J., Migueles J.H., Molina-García P., Henriksson H., Mena-Molina A., Martínez-Vizcaíno V. (2019). Role of physical activity and sedentary behavior in the mental health of preschoolers, children and adolescents: A systematic review and meta-analysis. Sports Med..

[62] Grao-Cruces A., Nuviala A., Fernández-Martínez A., Pérez-Turpin J.A. (2014). Association of physical self-concept with physical activity, life satisfaction and Mediterranean diet in adolescents. Kinesiology.

[63] Muros J.J., Cofre-Bolados C., Arriscado D., Zurita F., Knox E. (2017). Mediterranean diet adherence is associated with lifestyle, physical fitness, and mental wellness among 10-y-olds in Chile. Nutrition.

[64] Pulido J.J., Tapia-Serrano M.Á., Díaz-García J., Ponce-Bordón J.C., López-Gajardo M.Á. (2021). The relationship between students’ physical self-concept and their physical activity levels and sedentary behavior: The role of students’ motivation. Int. J. Environ. Res. Public. Health.

[65] Inchley J., Kirby J., Currie C. (2011). Longitudinal changes in physical self-perceptions and associations with physical activity during adolescence. Pediatr. Exerc. Sci..

[66] Palenzuela-Luis N., Duarte-Clíments G., Gómez-Salgado J., Rodríguez-Gómez J.Á., Sánchez-Gómez M.B. (2022). International Comparison of Self-Concept, Self-Perception and Lifestyle in Adolescents: A Systematic Review. Int. J. Public. Health.

[67] Fitzgerald N., Spaccarotella K. (2009). Barriers to a healthy lifestyle: From individuals to public policy—An ecological perspective. J. Ext..

[68] Benitez-Sillero J.d.D., Portela-Pino I., Morente Á., Raya-González J. (2023). Longitudinal relationships between physical fitness with physical self-concept and self-esteem in adolescents. Res. Q. Exerc. Sport..

[69] Cadenas-Sanchez C., Mena-Molina A., Torres-Lopez L.V., Migueles J.H., Rodriguez-Ayllon M., Lubans D.R., Ortega F.B. (2021). Healthier minds in fitter bodies: A systematic review and meta-analysis of the association between physical fitness and mental health in youth. Sports Med..

[70] Farren G.L., Zhang T., Gu X., Thomas K.T. (2018). Sedentary behavior and physical activity predicting depressive symptoms in adolescents beyond attributes of health-related physical fitness. J. Sport. Health Sci..

[71] Korczak D.J., Madigan S., Colasanto M. (2017). Children’s physical activity and depression: A meta-analysis. Pediatrics.

[72] Rieck T., Jackson A., Martin S.B., Petrie T., Greenleaf C. (2013). Health-related fitness, body mass index, and risk of depression among adolescents. Med. Sci. Sports Exerc..

[73] Greenleaf C.A., Petrie T.A., Martin S.B. (2010). Psychosocial variables associated with body composition and cardiorespiratory fitness in middle school students. Res. Q. Exerc. Sport..

[74] Schuch F.B., Vancampfort D., Sui X., Rosenbaum S., Firth J., Richards J., Ward P.B., Stubbs B. (2016). Are lower levels of cardiorespiratory fitness associated with incident depression? A systematic review of prospective cohort studies. Prev. Med..

[75] Barahona-Fuentes G., Huerta Ojeda Á., Romero G.L., Delgado-Floody P., Jerez-Mayorga D., Yeomans-Cabrera M.-M., Chirosa-Ríos L.J. (2023). Muscle Quality Index is inversely associated with psychosocial variables among Chilean adolescents. BMC Public Health.

[76] Delgado-Floody P., Gómez-López M., Caamaño-Navarrete F., Valdés-Badilla P., Jerez-Mayorga D. (2023). The Mediating Role of the Muscle Quality Index in the Relation of Screen Time and Abdominal Obesity with Health-Related Quality of Life in Chilean Schoolchildren. Nutrients.

[77] Redondo-Tebar A., Fatouros I.G., Martinez-Vizcaino V., Ruíz-Hermosa A., Notario-Pacheco B., Sanchez-Lopez M. (2021). Association between gross motor competence and health-related quality of life in (pre) schoolchildren: The mediating role of cardiorespiratory fitness. Phys. Educ. Sport. Pedagog..

[78] Kandola A., Ashdown-Franks G., Stubbs B., Osborn D., Hayes J. (2019). The association between cardiorespiratory fitness and the incidence of common mental health disorders: A systematic review and meta-analysis. J. Affect. Disord..

[79] Balsalobre F.J.B., Sánchez G.F.L., Suárez A.D. (2014). Relationships between physical fitness and physical self-concept in Spanish adolescents. Procedia-Soc. Behav. Sci..

[80] Dunton G.F., Schneider M., Graham D.J., Cooper D.M. (2006). Physical activity, fitness, and physical self-concept in adolescent females. Pediatr. Exerc. Sci..

[81] Tapia-Serrano M.A., Jorge M.-L., David S.-O., Mikel V.-S., Sanchez-Miguel P.A. (2021). Mediating effect of fitness and fatness on the association between lifestyle and body dissatisfaction in Spanish youth. Physiol. Behav..

[82] Haugen T., Ommundsen Y., Seiler S. (2013). The relationship between physical activity and physical self-esteem in adolescents: The role of physical fitness indices. Pediatr. Exerc. Sci..

[83] Rahman M.S., Helgadóttir B., Hallgren M., Forsell Y., Stubbs B., Vancampfort D., Ekblom Ö. (2018). Cardiorespiratory fitness and response to exercise treatment in depression. BJPsych Open.

[84] Silverman M.N., Deuster P.A. (2014). Biological mechanisms underlying the role of physical fitness in health and resilience. Interface Focus..

[85] Miller A.H. (2020). Beyond depression: The expanding role of inflammation in psychiatric disorders. World Psychiatry.

